# Recent Development in Partial Differential Equations and Their Applications

**DOI:** 10.1155/2014/243461

**Published:** 2014-06-25

**Authors:** Hossein Jafari, Chaudry M. Khalique, Dumitru Baleanu

**Affiliations:** ^1^Department of Mathematics, University of Mazandaran, Babolsar 47416-95447, Iran; ^2^International Institute for Symmetry Analysis and Mathematical Modelling, Department of Mathematical Sciences, North-West University, Mafikeng Campus, Private Bag Box X2046, Mmabatho 2735, South Africa; ^3^Department of Mathematics, Çankaya University, Balgat, 06530 Ankara, Turkey

In recent years, the partial differential equations, both fractional and integer orders, have been recognized as a powerful modeling methodology. They are inspired by problems which arise in diverse fields such as biology, fluid dynamics, physics, differential geometry, control theory, materials science, and engineering.

The purpose of this special issue is to report and review some recent developments in methods and applications of partial differential equations. The majority of the papers contained in this special issue are based on areas of research ranging from functional analytic techniques and singularity methods as well as numerical methods that are applied to both partial and ordinary differential equations. There are papers which deal with fractional partial differential equations and in addition papers analyzing equations that arise in engineering as well as classical and fluid mechanics.

This special issue contains the papers addressing the recent theoretical advances and experimental results on the topics such as operational calculus and inverse differential operators, global existence and energy decay rates for a Kirchhoff-type wave equation, nonsymmetric system of Keyfitz-Kranzer type, different methods for numerical solution, recent progress on nonlinear Schrödinger systems with quadratic interactions, upper semicontinuity of pullback attractors, numerical modeling, mean-variance portfolio selection, a novel iterative scheme, and the iterative methods of linearization.

The guest editors very much hope that the papers published in this special issue will be useful to a large community of researchers and will arouse further research in the topics presented as well as in the connected fields.

